# Supplementary material for: Genome-Wide Identification and Functional Evolution of NLR Gene Family in Capsicum annuum
Source: Curr Issues Mol Biol. 2025 Oct 21;47(10):867. doi: 10.3390/cimb47100867 (PMC12562843; doi:10.3390/cimb47100867)
Supplement: Supplementary file 1 [file cimb-47-00867-s001.zip › cimb-3872540-supplementary.pdf]

Table S1 Primers used for RT-qPCR.

Sequence ID	Primer ID	Primer Sequence
CaUBI-3	CaUBI-3-F	GTCCATCTGCTCTCTGTTG
	CaUBI-3-R	CACCCCAAGCACAATAAGAC
Caz09g03770	Caz09g03770-F	TGGCGAAGAATGGCATCCA
	Caz09g03770-R	GAGGCGCTCAAGGACAGG
Caz03g40070	Caz03g40070-F	GCATGCCCGGTCTTGGTA
	Caz03g40070-R	TCCCGCTCTTGGCTGTTG
Caz10g21150	Caz11g00620-F	GCCCAGTCGCAGAACACT
	Caz11g00620-R	CTGCACCCCGCTTCTGAA
Caz10g20900	Caz10g20900-F	TTGCGCGACAGATTGGGT
	Caz10g20900-R	AAGTTTCTCCGGGCAGCC

Table S2 The NLR gene related information involved in this study.

Gene ID	Length(aa)	Molecular Weight (Da)	pI
Caz01g09900	856	96390.3	7.5
Caz01g09970	779	88473.5	7.9
Caz01g09980	678	77426.6	8.4
Caz01g19520	1346	153255	5.8
Caz01g22900	734	84459.4	8.4
Caz01g23590	744	84526.6	6.1
Caz01g24300	1356	153477.6	6
Caz01g24620	751	85467.2	6
Caz01g32120	664	76987.4	5.9
Caz01g32550	881	100910	8.8
Caz01g35160	883	100805.7	8.6
Caz01g37240	836	95385.5	6.5
Caz01g37340	944	107087	7.5
Caz01g37350	1627	184870.9	6.3
Caz01g37380	1560	176902.7	6.3
Caz01g37400	1625	184613.6	6.4
Caz01g37410	943	106890.8	7.1
Caz01g37560	939	107145.5	6.2
Caz01g37940	1019	115629.6	6.4
Caz01g38720	487	55576.3	6
Caz01g39390	1604	184240.9	7.6
Caz01g39530	927	106322.5	8.5
Caz01g41000	1518	172355.3	6
Caz01g41040	1426	162027.7	6.9
Caz01g41060	770	87699.2	5.9
Caz01g41070	995	113283.9	5.9
Caz01g41090	729	82319.4	6
Caz01g41650	1615	187807.4	6
Caz02g12040	946	108462.2	8.6
Caz02g12080	940	107312	8.2
Caz02g12090	573	65588.2	8.6
Caz02g13420	1223	140726.4	6.2
Caz02g20380	1244	139841	6.5
Caz02g20420	1059	119635.5	8.1
Caz02g21920	821	93534.7	5.7
Caz02g21940	892	101409	5.6
Caz02g21980	930	105866.1	5.8
Caz02g23070	848	96486.1	6.1
Caz02g27530	852	97033.7	6.2
Caz02g27540	850	96874	6

Caz03g17090	715	82233.8	6.5
Caz03g30170	745	85569.5	5.8
Caz03g35520	1148	132568.1	6.6
Caz03g36190	1330	153587.6	5.8
Caz03g36340	964	110610.4	6.5
Caz03g36490	1093	124100.7	7.3
Caz03g36560	800	91554.3	9
Caz03g36610	562	64518.6	6.2
Caz03g36930	771	87550.6	6.1
Caz03g36950	588	67063.2	6.4
Caz03g37000	829	95074.8	6.6
Caz03g37050	669	76770.1	9
Caz03g37840	1223	140985.1	8.5
Caz03g38120	776	87338.8	5.5
Caz03g40050	879	100949.1	8.3
Caz03g40070	802	91803.2	8.6
Caz03g40090	817	93388.2	8.7
Caz03g40550	788	89710.5	8.3
Caz03g41600	732	84922.8	5.4
Caz03g41640	1263	146554.2	8.3
Caz03g41650	1141	131549.1	6.6
Caz03g41680	703	80086.6	7
Caz03g41690	1240	143384.3	6.3
Caz03g41750	915	105307.6	7.6
Caz03g41760	969	110672.8	6.5
Caz03g41780	679	78153.7	6.2
Caz03g41800	1137	130528.3	5.8
Caz03g41900	985	114945	4.7
Caz04g00040	851	97227.7	8.7
Caz04g00080	692	79321.7	6
Caz04g00090	779	89104.5	8.5
Caz04g04400	831	94957.1	5.8
Caz04g06300	877	101053.5	6
Caz04g06310	1706	195459	6.6
Caz04g11150	853	98081.3	5.4
Caz04g21160	1217	138558.5	6
Caz04g22510	934	106817.4	7.8
Caz05g00440	827	94750.9	7.2
Caz05g01980	1228	138588	6.1
Caz05g02000	849	96475.1	7
Caz05g02920	1328	150802.3	6.1
Caz05g03280	893	102446.2	8.1
Caz05g03300	1030	118191	6.2
Caz05g03360	869	100117.2	5.9

Caz05g03390	705	81438.3	6.9
Caz05g04610	722	82823.3	8.4
Caz05g06430	860	98644	6.7
Caz05g06440	903	104375.6	6
Caz05g06650	673	76511.7	8.7
Caz05g07340	1227	140189.7	8.1
Caz05g07520	855	99001.6	7.8
Caz05g07630	832	94914.6	6.7
Caz05g07850	890	103719.7	8.9
Caz05g14280	513	58907.3	7.9
Caz05g14840	776	90225.7	7.2
Caz05g16380	912	104590.4	6.2
Caz05g16670	769	87966.2	5.6
Caz05g16720	762	87672.3	5.2
Caz05g17480	1190	135980.4	5.2
Caz05g19310	1225	140914.8	5.2
Caz05g19320	1202	137929.2	5.4
Caz05g19370	1683	192466.6	5.1
Caz05g19410	1326	152146.4	5.8
Caz05g19440	1242	142280.8	6.2
Caz05g19510	1763	202057.6	5.1
Caz05g19520	1292	148650.6	4.9
Caz05g19530	1258	144621.6	5.1
Caz05g19550	1253	143829.8	4.8
Caz05g19570	1298	148882.2	4.9
Caz05g19980	1134	130278.8	5.7
Caz05g20940	1182	135240.2	5.5
Caz05g20950	573	65525.4	5.4
Caz05g21030	538	61530.2	5.7
Caz06g14960	1152	132274.7	8.3
Caz06g14970	1497	171065.3	6.8
Caz06g14990	1550	177760.5	8.1
Caz06g25760	1008	115861.8	5.1
Caz06g25770	1076	124243.2	5.3
Caz06g27510	991	113817.7	5
Caz06g28240	839	95971.6	5.5
Caz06g28260	580	66920.6	6.4
Caz06g28360	820	94508.7	5.2
Caz06g28410	1168	134188.6	4.9
Caz06g28420	1010	116202.1	4.8
Caz06g28570	1139	131639	5.5
Caz06g28880	1082	124233	5.3
Caz06g28890	1050	120838.5	5.4
Caz06g28970	1104	127532.7	5.9

Caz06g28980	967	112071.3	5.4
Caz06g29000	1356	155306.2	5.5
Caz06g29600	986	113003.5	5.3
Caz06g29650	982	112351	5.2
Caz06g29950	927	106513.1	5.2
Caz06g30060	852	97811.1	5.8
Caz07g00070	969	110634.7	6.1
Caz07g00080	1705	194332.9	5
Caz07g00270	859	98863.2	6.7
Caz07g02390	964	109960.9	5.8
Caz07g02430	824	94914.2	8.3
Caz07g06100	708	82526.4	6
Caz07g11710	870	99481.4	8.2
Caz07g14800	803	92194.9	6.1
Caz07g14830	869	98410.9	5.8
Caz07g14840	882	100542.3	5.9
Caz07g14870	844	96789.7	6.2
Caz07g14900	530	60852.4	7.1
Caz07g14910	515	59314	9.5
Caz07g15050	919	103494.9	8.2
Caz07g20150	1281	147580.4	5.5
Caz07g21640	1015	118174.2	7
Caz07g25030	1107	126230.5	5.4
Caz07g25060	804	91519.2	5.5
Caz08g00580	883	101630.9	6.9
Caz08g01200	741	85787.2	6.8
Caz08g01210	572	65823.7	6
Caz08g01220	889	103596.3	8.2
Caz08g01230	884	102918.7	6.7
Caz08g14490	1228	140289.3	8.4
Caz08g15790	1431	162751.7	6.8
Caz08g15800	1043	118852.6	8.1
Caz08g15820	1174	133475.9	7.3
Caz08g19150	1178	133338.6	6.2
Caz08g19160	1187	134248	6.5
Caz09g00020	778	89236.5	5.8
Caz09g00030	826	95120.8	5.1
Caz09g00210	1143	129778.3	8.7
Caz09g00460	785	90343.8	6.3
Caz09g01230	868	98998.7	5.7
Caz09g01360	677	77909.7	8.1
Caz09g01420	1112	126702.1	5.8
Caz09g01460	1120	128321.3	5.5
Caz09g01530	627	71536.3	6

Caz09g01540	1649	189535.2	5.4
Caz09g01670	1152	131672.6	5.5
Caz09g01710	1546	176244	5.4
Caz09g01720	1540	175450.4	5.7
Caz09g01780	725	82381.7	5.4
Caz09g02010	849	96187.6	5.2
Caz09g02310	950	109110.7	5.6
Caz09g02320	994	113587.5	4.8
Caz09g02330	1048	120762.6	5.4
Caz09g02350	621	71873.8	5.3
Caz09g02360	2270	260534.8	5.4
Caz09g02470	940	108393.1	5.5
Caz09g02480	1181	135104.9	5.5
Caz09g02490	907	103568.8	6
Caz09g02530	925	106434	5.2
Caz09g02540	871	99971.6	5.1
Caz09g02550	951	108740.7	5.5
Caz09g02560	867	99229.6	5.2
Caz09g02570	1033	118955	6.2
Caz09g02580	1780	203750.7	5.3
Caz09g02850	885	102146.9	7.6
Caz09g02920	666	76006.5	8.6
Caz09g03450	639	73532.9	8
Caz09g03510	839	96649.4	6.2
Caz09g03580	670	76344.4	5.9
Caz09g03710	754	87039.8	5.5
Caz09g03740	684	78062	5.2
Caz09g03750	755	85814.5	8.2
Caz09g03770	669	77608.2	7.2
Caz09g03780	1184	135480.7	5.2
Caz09g03810	532	60622.6	6.8
Caz09g03820	1253	142777.1	5.7
Caz09g03850	715	81942.7	5.6
Caz09g03960	911	104904.7	5.7
Caz09g05530	850	97864.1	6.7
Caz09g05540	670	76937.2	7.8
Caz09g06440	888	101967.2	7.9
Caz09g06570	923	105948.3	6.4
Caz09g06820	966	110285.5	5.1
Caz09g13560	734	83734	6.6
Caz09g17700	1370	157245.5	5.7
Caz09g19160	807	93712.3	7.3
Caz09g19740	928	106875.5	7.1
Caz10g01200	643	74167.7	6.7

Caz10g03310	805	92229	8.4
Caz10g03320	1731	198677.6	6.4
Caz10g03340	1017	116904.2	8.2
Caz10g11630	1241	142010.1	6.3
Caz10g15990	1254	144353.4	5.8
Caz10g19600	1300	147343	6.4
Caz10g19780	1229	139637.7	7.8
Caz10g20470	1244	141581.6	5.4
Caz10g20500	961	108632.8	7.2
Caz10g20510	1120	127057.9	7.2
Caz10g20610	752	85124.8	6
Caz10g20620	770	87835.2	5.5
Caz10g20830	807	93276.4	6
Caz10g20870	1447	163820.6	5.6
Caz10g20880	988	112198.6	5.5
Caz10g20890	746	86050.4	5.9
Caz10g20900	1601	182493.5	6.1
Caz10g21110	1242	140641.2	5.1
Caz10g21150	1086	123577.5	6
Caz10g21160	1560	176855.1	5.3
Caz10g21190	1002	114721.6	5.8
Caz11g00620	1338	151544	5.8
Caz11g00630	1318	149759.8	5.8
Caz11g00640	635	72597.1	8.8
Caz11g00660	1092	124363.9	5.4
Caz11g00680	950	105836.9	7.9
Caz11g01620	878	100537.3	8.9
Caz11g03440	1270	144031	5.7
Caz11g03450	1591	181088.1	6.6
Caz11g04480	820	93282.3	5.2
Caz11g04650	578	66076.6	6
Caz11g12540	1347	153889	6.3
Caz11g14710	1278	144177.2	7
Caz11g14730	1212	136598.4	6.2
Caz11g16400	1189	136687.4	8.3
Caz11g16830	764	88012.4	6.6
Caz11g16850	1078	123774.1	5.8
Caz11g16860	1583	179898	6.1
Caz11g16900	1717	196050.8	6.6
Caz11g16920	1231	140857.2	5.9
Caz11g17790	1191	136900.1	6.5
Caz11g18650	772	89138.1	6.2
Caz11g19950	913	105158.6	6.3
Caz11g20340	880	100667	8.4

Caz11g20390	880	100729.9	8.1
Caz11g21320	604	70622.6	5.5
Caz11g21340	753	87658.8	6.1
Caz11g21370	705	81439.8	7.2
Caz11g21460	1348	154793.6	8.3
Caz11g21470	888	101572.1	7.7
Caz11g21490	897	101975.4	8.2
Caz11g21510	888	102447.7	8
Caz11g23340	1105	125526.4	7.3
Caz11g23360	896	103112.8	5.8
Caz12g03560	1312	151790.4	6.1
Caz12g03610	1227	142328.7	6.2
Caz12g03620	1242	144491.9	6.7
Caz12g03630	1290	149008.4	6.2
Caz12g03640	1116	129155.8	6
Caz12g04280	1194	136658.6	8
Caz12g04290	960	109851.3	7.5
Caz12g04380	957	111131.7	8.8
Caz12g06030	1120	128691.2	6.2
Caz12g06270	781	90047.9	6.6
Caz12g07040	773	88112.7	8.3
Caz12g07860	1375	157207.5	8.7
Caz12g07980	1362	157058.4	7.2
Caz12g08320	1233	142926.7	5.8
Caz12g20760	982	113481.9	6.1
Caz12g21860	1175	134322.3	6.1
Caz12g23540	901	102255.6	6.7
Caz12g25100	1099	126683.3	6.3

Table S3 Intraspecific Ka values of pepper NLR genes

Seq_1	Seq_2	Ka	Ks	Ka/Ks
Caz01g09970.1	Caz01g09980.1	0.16	0.35	0.44
Caz01g41060.1	Caz01g41070.1	0.11	0.18	0.59
Caz02g12080.1	Caz02g12090.1	0.06	0.20	0.30
Caz02g27530.1	Caz02g27540.1	0.06	0.18	0.32
Caz03g40070.1	Caz03g40090.1	0.17	0.40	0.43
Caz03g41640.1	Caz03g41650.1	0.29	0.42	0.68
Caz04g06300.1	Caz04g06310.1	0.11	0.32	0.35
Caz05g06430.1	Caz05g06440.1	0.23	0.39	0.59
Caz05g19520.1	Caz05g19530.1	0.11	0.18	0.59
Caz06g14960.1	Caz06g14970.1	0.04	0.09	0.47
Caz07g14830.1	Caz07g14840.1	0.01	0.01	0.73
Caz08g01200.1	Caz08g01210.1	0.07	0.12	0.58
Caz08g01210.1	Caz08g01220.1	0.14	0.24	0.61
Caz08g01220.1	Caz08g01230.1	0.15	0.30	0.51
Caz08g15790.1	Caz08g15800.1	0.47	1.85	0.25
Caz08g19150.1	Caz08g19160.1	0.47	2.10	0.23
Caz09g00020.1	Caz09g00030.1	0.18	0.21	0.84
Caz09g01710.1	Caz09g01720.1	0.01	0.01	1.07
Caz09g02320.1	Caz09g02330.1	0.09	0.14	0.66
Caz09g02350.1	Caz09g02360.1	0.07	0.09	0.79
Caz09g05530.1	Caz09g05540.1	0.08	0.11	0.72
Caz10g20870.1	Caz10g20880.1	0.17	0.21	0.83
Caz11g00620.1	Caz11g00630.1	0.05	0.05	0.97
Caz11g00630.1	Caz11g00640.1	0.12	0.25	0.49
Caz11g03440.1	Caz11g03450.1	0.14	0.27	0.50
Caz12g03610.1	Caz12g03620.1	0.11	0.09	1.31
Caz12g03620.1	Caz12g03630.1	0.26	0.45	0.57
Caz12g03630.1	Caz12g03640.1	0.25	0.40	0.62
Caz12g04280.1	Caz12g04290.1	0.03	0.05	0.67

Table S4 Comparison of nonsynonymous substitution rates of NLR orthologous gene pairs between pepper and tomato.

Seq_1	Seq_2	Ka	Ks	Ka/Ks
Caz01g32550.1	Solyc01g090430.3.1	0.10	0.31	0.31
Caz01g22900.1	Solyc01g073985.1.1	0.07	0.22	0.30
Caz01g23590.1	Solyc01g087200.3.1	0.05	0.26	0.21
Caz01g09900.1	Solyc08g076000.4.1	0.09	0.20	0.45
Caz01g39390.1	Solyc08g005500.4.1	0.21	0.37	0.56
Caz01g39530.1	Solyc08g005440.3.1	0.09	0.31	0.29
Caz02g12040.1	Solyc02g070750.2.1	0.09	0.27	0.35
Caz02g20380.1	Solyc02g082050.3.1	0.13	0.59	0.23
Caz02g23070.1	Solyc02g084890.3.1	0.04	0.21	0.19
Caz02g27530.1	Solyc02g090380.4.1	0.09	0.27	0.34
Caz02g27530.1	Solyc04g079420.3.1	0.71	2.50	0.28
Caz03g40050.1	Solyc09g092280.1.1	0.22	0.49	0.45
Caz03g41690.1	Solyc09g098100.4.1	0.18	0.33	0.54
Caz03g41900.1	Solyc09g098210.3.1	0.21	0.53	0.39
Caz04g04400.1	Solyc04g079420.3.1	0.03	0.27	0.12
Caz05g01980.1	Solyc04g015630.2.1	0.09	0.26	0.35
Caz05g03280.1	Solyc04g011980.1.1	0.24	0.39	0.62
Caz05g03300.1	Solyc04g011960.2.1	0.24	0.39	0.62
Caz05g03360.1	Solyc04g011890.1.1	0.13	0.35	0.38
Caz05g06430.1	Solyc04g009240.2.1	0.24	0.42	0.58
Caz05g06440.1	Solyc04g009110.1.1	0.19	0.35	0.53
Caz05g07340.1	Solyc04g008690.3.1	0.08	0.24	0.32
Caz05g00440.1	Solyc04g039980.3.1	0.06	0.20	0.29
Caz05g19530.1	Solyc05g053980.1.1	0.30	0.56	0.53
Caz05g19570.1	Solyc05g054020.3.1	0.34	0.60	0.57
Caz06g14960.1	Solyc06g065120.1.1	0.24	0.55	0.45
Caz06g29650.1	Solyc06g008368.2.1	0.26	0.48	0.55
Caz06g29600.1	Solyc06g008765.2.1	0.22	0.48	0.46
Caz07g14800.1	Solyc07g053010.3.1	0.08	0.33	0.24
Caz08g14490.1	Solyc01g113620.3.1	0.07	0.20	0.34
Caz08g19150.1	Solyc01g106410.3.1	0.05	0.18	0.29
Caz09g13560.1	Solyc09g005290.1.1	0.08	0.25	0.30
Caz09g06570.1	Solyc12g005970.1.1	0.11	0.28	0.39
Caz09g02010.1	Solyc12g009415.2.1	0.22	0.46	0.48
Caz10g15990.1	Solyc09g009960.3.1	0.71	1.29	0.55
Caz10g03310.1	Solyc10g008220.4.1	0.08	0.26	0.30
Caz10g03320.1	Solyc10g008230.3.1	0.19	0.39	0.48
Caz10g20830.1	Solyc10g078830.2.1	0.44	1.52	0.29
Caz10g21150.1	Solyc10g079020.1.1	0.24	0.47	0.50
Caz10g20610.1	Solyc10g079020.1.1	0.19	0.27	0.71

Caz10g20470.1	Solyc10g055180.1.1	0.55	0.83	0.66
Caz10g20610.1	Solyc10g055170.1.1	0.30	0.62	0.48
Caz10g20620.1	Solyc10g055120.1.1	0.32	0.49	0.65
Caz10g20870.1	Solyc10g054970.1.1	0.31	0.41	0.74
Caz10g21110.1	Solyc10g054960.1.1	0.32	0.42	0.75
Caz10g21190.1	Solyc10g054940.1.1	0.31	0.46	0.67
Caz11g23340.1	Solyc02g091420.3.1	0.24	1.85	0.13
Caz11g21470.1	Solyc04g007030.4.1	0.43	1.52	0.28
Caz11g14710.1	Solyc05g006620.3.1	0.07	0.19	0.37
Caz11g16400.1	Solyc05g007350.3.1	0.09	0.24	0.38
Caz11g16860.1	Solyc05g007610.2.1	0.26	0.49	0.53
Caz11g17790.1	Solyc05g013280.4.1	0.64	1.89	0.34
Caz11g17790.1	Solyc05g012740.2.1	0.16	0.25	0.64
Caz11g23340.1	Solyc05g008070.4.1	0.26	0.57	0.46
Caz11g21320.1	Solyc05g009740.1.1	0.34	1.10	0.31
Caz11g21460.1	Solyc05g009630.4.1	0.07	0.71	0.10
Caz11g16400.1	Solyc05g007610.2.1	0.35	0.74	0.47
Caz11g12540.1	Solyc05g013250.3.1	0.22	0.56	0.40
Caz11g00680.1	Solyc06g074930.3.1	0.38	1.88	0.20
Caz11g00620.1	Solyc11g069620.3.1	0.19	0.35	0.56
Caz11g00640.1	Solyc11g161760.1.1	0.30	0.94	0.32
Caz11g00620.1	Solyc11g069920.1.1	0.23	0.36	0.66
Caz11g01620.1	Solyc11g069020.4.1	0.11	0.42	0.26
Caz12g23540.1	Solyc11g010160.1.1	0.10	0.34	0.31
Caz12g25100.1	Solyc11g011350.4.1	0.20	0.34	0.59
Caz12g04290.1	Solyc12g097000.2.1	0.13	0.41	0.31
Caz12g04380.1	Solyc12g096920.1.1	0.07	0.46	0.16
Caz12g06030.1	Solyc12g094650.3.1	0.12	0.27	0.46

Table S5 List of identified motifs and their E-values.

Motif name	Sequence	E value
motif 1	VVSIVGMGGVGKTTLAKKVYN	$4.6e^{-464}$
motif 2	CKGLPLAIVVIAGVL	$2.2e^{-262}$
motif 3	LKGKRYLIVLDDVWD	$9.3e^{-235}$
motif 4	MKVJELSYBHLPDHLKPCFLY	$3.6e^{-211}$
motif 5	NGSRIIJTTRNKEVA	$3.9e^{-200}$
motif 6	PLELRLLSEEESWELFRKKAF	$3.7e^{-204}$
motif 7	CKMHDLVRDLC	$2.1e^{-170}$
motif 8	YPEDYEIPVDELIRLWIAEGL	$3.1e^{-213}$
motif 9	LPSSISNLWNLETLI	$2.2e^{-167}$
motif 10	HFDVRAWVTVSQZYD	$2.1e^{-158}$
