# Supplementary material for: Genome-Wide Identification and Functional Evolution of NLR Gene Family in Capsicum annuum
Source: Curr Issues Mol Biol. 2025 Oct 21;47(10):867. doi: 10.3390/cimb47100867 (PMC12562843; doi:10.3390/cimb47100867)
Supplement: Supplementary file 1 [file cimb-47-00867-s001.zip › Supplementary Figures.pdf]

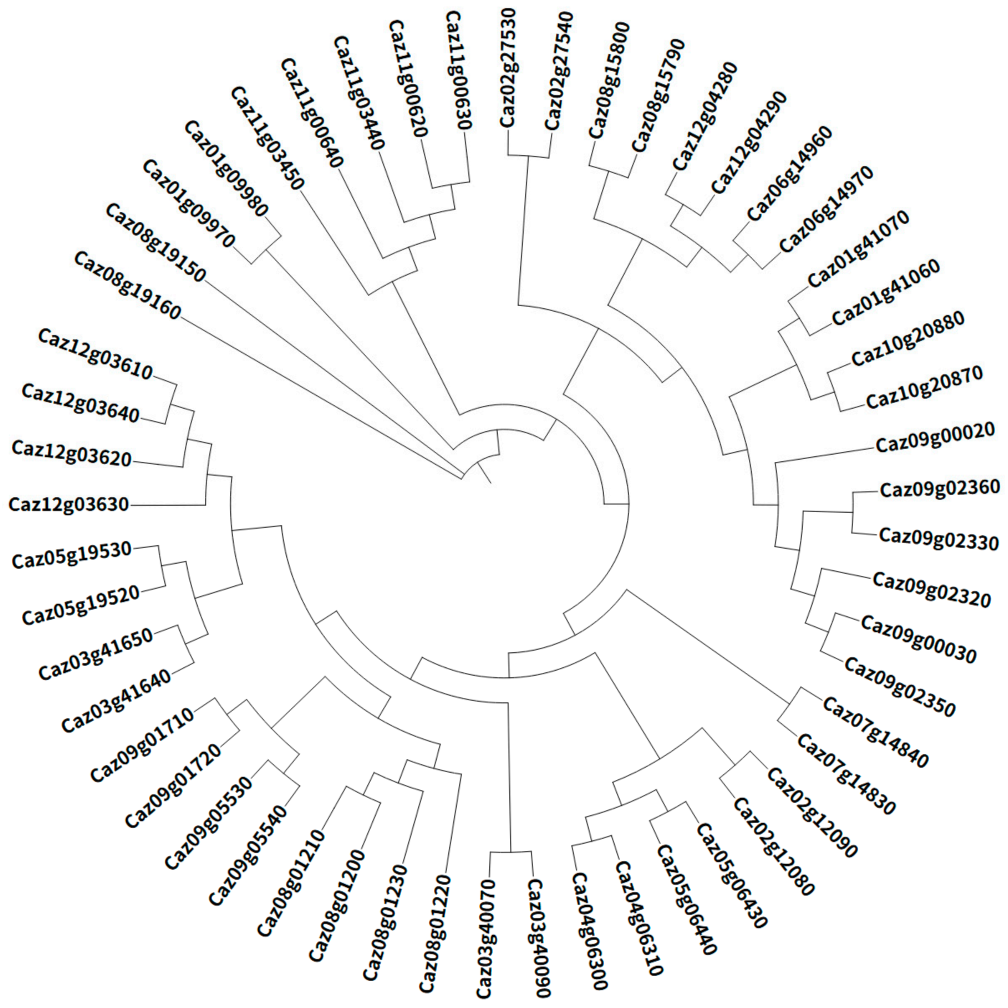

Figure S1 Phylogenetic analysis of 53 tandemly duplicated NLR genes in pepper.

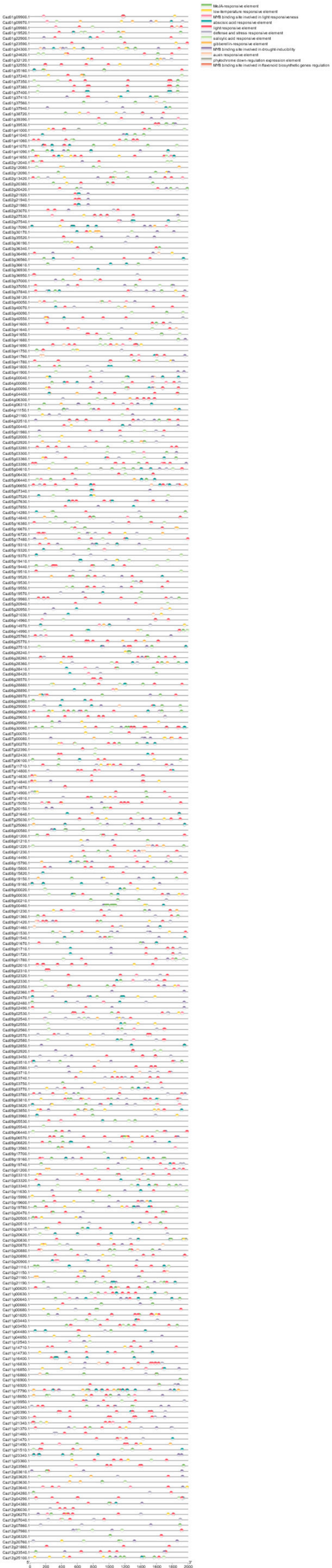
MeJA-responsive element
low-temperature responsive element
MYB binding site involved in light responsiveness
abscisic acid responsive element
light responsive element
defense and stress responsive element
salicylic acid responsive element
gibberellin-responsive element
MYB binding site involved in drought-inducibility
auxin responsive element
phytochrome down-regulation expression element
MYB binding site involved in flavonoid biosynthetic genes regulation
0
200
400
600
800
1000
1200
1400
1600
1800
2000

Figure S2 *Cis*-regulatory elements in the 2-kb upstream region of 228 NLRs coding sequences. Rounded rectangles with different colors indicate different cis-acting elements.

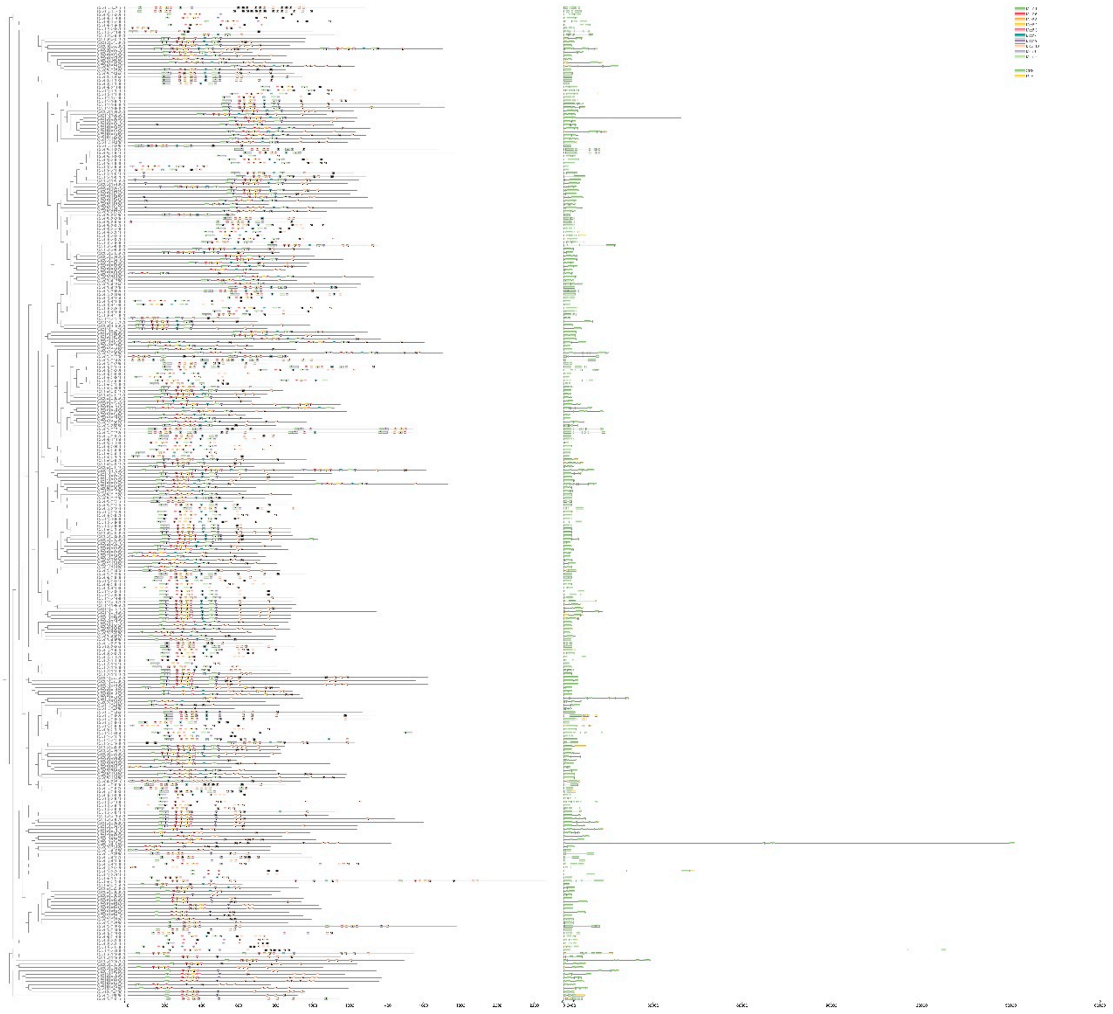

Figure S3 The phylogenetic relationships of the NLRs genes constructed with bootstrap values of 1000 replicates. Conserved motifs and domains of NLRs genes were analyzed using MEME (Left). The gene structures of the NLRs genes analyzed using TBtools. CDS and UTRs are colored with green and yellow boxes, respectively, where black lines represent introns (Right).
